# Bayesian Inference of Sex-Specific Mortality Profiles and Product Yields from Unsexed Cattle Zooarchaeological Remains

**DOI:** 10.1007/s10816-025-09749-x

**Published:** 2025-11-06

**Authors:** Yoan Diekmann, Rosalind E. Gillis, Ziye Lu, Anna Rudzinski, Maria De Iorio, Mark G. Thomas

**Affiliations:** 1 Research Department of Genetics, Evolution and Environment, University College London, London, WC1E 6BT UK; 2 Deutsches Archäologisches Institut, Referat Naturwissenschaften, Berlin, 14195 Germany; 3 Interdisciplinary Centre for Archaeology and Evolution of Human Behaviour (ICArEHB), Universidade do Algarve, Faro, 8005-139 Portugal; 4 Department of Paediatrics, Yong Loo Lin School of Medicine, National University of Singapore, Singapore, Singapore; 5 A*STAR Institute for Human Development and Potential, Singapore, Singapore; 6 UCL Genetics Institute, University College London, London, WC1E 6BT UK

**Keywords:** Age-at-death, Bayesian inference, MCMC, Paleoeconomics, Zooarchaeology

## Abstract

Zooarchaeological age-at-death profiles for domesticated ruminants can be inferred from tooth eruption, replacement, and wear. These profiles contain important information on slaughter management and have been used informally to infer the goals of past husbandry strategies. In principle, sex-specific survival curves could inform on various productivity parameters, including herd growth rates and sustainability, milk and meat yields, macronutrient and calorie yields, and feed consumed. Knowledge of these parameter values would allow identification of differences in husbandry economics in different archaeological contexts. However, archaeological age-at-death profiles are rarely sex-specific and are often derived from small sample sizes. As such, challenges remain in inferring sex-specific survival curves using explicit models that account for sampling uncertainty. We present a Bayesian inference approach for inferring sex-specific survival curves from unsexed cattle zooarchaeological age-at-death profiles that can accommodate data from any combination of age class boundaries. Our approach relies on the assumption that asymmetric sex-specific slaughter leads to a change in sex ratio over time, which we inform from slaughter practices in modern unimproved cattle herds. By combining inferred sex-specific archaeological survival curves with ethnographic productivity data from modern unimproved cattle, we are able to estimate herd growth rate, milk and meat yields, macronutrient and calorie yields, and feed consumed per animal. We apply our approach to zooarchaeological age-at-death profiles previously proposed to prioritise milk or meat production and to a set of profiles from ten Neolithic sites located across Europe. We infer that there was scope for improvement in prehistoric slaughter management.

**Supplementary Information:**

The online version contains supplementary material available at 10.1007/s10816-025-09749-x.

## Introduction

Patterns of tooth eruption and wear can be used to estimate age-at-death in humans (Manjunatha & Soni, 2014) and other animals (Cornevin & Lesbre, 1894). In archaeozoology, bovid tooth eruption through the mandible or maxilla bone, as well as the development, wear, and replacement of teeth, has been used to estimate age-at-death assigned to fixed age classes of varying number and temporal widths (Ducos, 1968; Grigson, 1982; Habermehl, 1975; Halstead, 1985; Hambleton, 1999; Higham, 1967; Jones & Sadler, 2013b; Legge, 1992; O'Connell, 1991). Because there is better age resolution for juvenile animals, these age classes are typically narrower over the first few years of life than later (Gerbault *et al.*, 2016). Examination of modern cattle has revealed good agreement between estimated and true ages based on tooth eruption and wear (Jones & Sadler, 2013a; Legge, 1992). In comparison to post-cranial age determination, dental eruption, replacement, and wear are generally considered the best archaeozoological indicators of past slaughter management practices (Vigne & Helmer, 2007).

Recently, statistical methods for the analysis of archaeozoological age-at-death data have been proposed that accommodate sampling uncertainty and have been used to make robust comparisons of different assemblages (Gerbault *et al.*, 2016; Gillis *et al.*, 2017; Millard, 2006; Price *et al.*, 2016; Timpson *et al.*, 2018; Weaver *et al.*, 2011). The mortality profile of a managed herd will have important implications for herd sustainability, meat and milk yields, and the quantities of feed consumed. This has led to considerable interest in inferring the goals of past husbandry strategies using archaeozoological age-at-death profiles (Halstead, 1998; Helmer, 1992; Helmer *et al.*, 2005; Higham, 1967; Payne, 1973; Redding, 1981; Vigne, 1988; Vigne & Helmer, 2007), in particular, the relative importance of milk and meat production (Gillis *et al.*, 2017; Helmer *et al.*, 2005; Legge, 1992).

For sheep and goats, Payne (1973) proposed three model (*i.e.* idealised) kill-off profiles, each based on 9 age classes; one optimized for meat yield, one optimized for milk yield, and one designed to maximize wool/fleece production. Redding (1981) proposed two sheep and goat model kill-off profiles, each based on 10 age classes; one optimized for energy yield and one designed to maximize herd security (*i.e.* herd growth rate, here referred to as sustainability). Based on different combinations of observed preferential culling age practices in modern stock in southeastern France and Iran, Vigne and Helmer (2007) defined five archaeological sheep and goats age-at-death profiles, each consisting of the expected proportions of teeth within 7 age classes. However, with the exception of one sex-specific model kill-off profile, based on observations of modern European dairy herds (McGrory *et al.*, 2012), equivalent idealized age-at-death patterns are not available for cattle. Based on observations of traditional breeds, where the presence of calves is necessary to stimulate the let-down of milk, males would be slaughtered post-lactation (Peške, 1994). This type of mortality profile was identified at the site of Bercy (Middle Neolithic, France, (Tresset, 1996)) and Popină Borduşani (Eneolithic, Romania) and confirmed via stable isotopic analysis (Balasse & Tresset, 2002; Gillis *et al.*, 2013). For intensive milking strategies, such as those seen in modern dairy production, male calves are removed at birth and either slaughtered or raised for meat production. This was proposed to be the situation at the Bronze Age site of Grimes Graves (Legge, 1992), where calves younger than 6 months dominate the slaughter profile. These sites have been used as models for post-lactation and intensive milk management (Gillis *et al.*, 2017).

Reliable inference of herd milk yield, as well as herd sustainability, requires sex-specific mortality profiles. While sexing of animal remains is possible, it is only possible with large assemblages and therefore is more challenging—and hence rarer—than age-at-death estimation in Neolithic assemblages, and usually restricted only to post-cranial elements of adults (Davis *et al.*, 2018). If sex-specific mortality profiles were available, they could be combined with information on animal growth trajectories, birth rates, and monthly milk and meat yields at different ages to estimate herd growth rates and lifetime milk, meat, macronutrient, and calorie yields. Furthermore, estimates of the costs of keeping animals at different ages (for example, by estimating feed consumed) would also permit estimation of the economic efficiency of various past slaughter management practices in terms of calorie production.

Data from archaeological (McGrory *et al.*, 2012) and modern unimproved cattle (Amanor, 1995; Ducrotoy *et al.*, 2016; Ladan, 1992; Pullan, 1979; Trail *et al.*, 1980b; Wagenaar *et al.*, 1986; Wilson, 1986) herds indicate sex asymmetries in slaughter patterns, with male animals typically killed more frequently at younger ages (see Supp. Table 1). Such strategies make sense since females are required for reproduction and milk production, which they continue after they reach sexual maturity, whereas only a few males are required for reproduction, and returns on their meat yields in terms of feed consumed diminish as they approach full adult weight. A model of sex-specific survival through time, in combination with unsexed archaeological kill-off profiles, would provide information on sex-specific mortality at different ages.

We present a Bayesian approach to estimating sex-specific mortality profiles by combining unsexed archaeological cattle kill-off data with sex-specific slaughter patterns in modern unimproved cattle (*i.e.* cattle that are part of a low-input grazing system, raised on pastures that have not been significantly altered or improved with fertilisers, pesticides, or other intensive farming practices). Full posterior inference is performed using Markov-chain Monte-Carlo (MCMC) methods. The overall structure of the algorithm is a Gibbs sampler with Metropolis-Hastings (Hastings, 1970) and Adaptive Rejection Sampling (Gilks & Wild, 1992) steps. MCMC methods allow drawing samples from the posterior distribution of the parameters of interest, which, in combination with ethnographic information on unimproved cattle birth rates, milk, meat and offal yields, and feed consumption, by age, can be used to derive predictions of the economic efficiency and macronutrient outputs of prehistoric herds, given unsexed kill-off data. Our approach can accommodate any combination of number and duration of age classes, as well as uncertainty on assignment of individuals to different age classes, as determined by archaeozoologists, enabling comparison of inferences from otherwise incompatible data.

## Materials and Methods

### Archaeological Data

Archaeological kill-off profiles are usually reported either as ‘number of elements’ (*i.e.* teeth) or ‘minimum number of individuals’ (MNI). The relationship between these statistics is poorly characterised. However, we elected only to examine archaeological sites where MNI estimates were available.

We considered ten MNI profiles from ten Neolithic sites (Gillis *et al.*, 2016, 2017); five profiles with the smallest total number of individuals (Polgar-Piócási-dűlo, Rosheim, Stephansposching, Těšetice-Kyjovice, and Windmill Hill) and the five profiles with the largest total number of individuals (Apc-Berekalja, La Draga, Trasano I, Füzseabony-Gubakút, and Mold) were selected from a larger dataset (see Supp. Table 3). These datasets are characterized by various levels of uncertainty (due to different sample size), which is fully captured and accounted for by the proposed approach. The age-at-death estimates from the ten Neolithic sites were determined according to Legge (1992). Both isolated teeth and fragmented mandibles were considered; we did not include third deciduous premolar and third premolar in the analysis. To calculate the MNI estimates, we first calculated the total left and right using the raw age assigned data where available and took the one with the largest value. Next, we considered isolated teeth and ensured they were not represented by other teeth. Ageable material from early farming sites is highly fragmented, and therefore, MNI counts may be shared across age classes, particularly for adults. We also compared parameter estimates from a set of kill-off profiles, previously used as references (Gillis *et al.*, 2017) for the following strategies: intensive milk (Grimes Graves, Bronze Age, UK (Legge, 1992)); post-lactation associated with mixed dairy and meat (Popina Bordusani, Eneolithic, Romania Bréhard & Bălăşescu, 2012; Gillis *et al.*, 2013); Bercy, Middle Neolithic, France (Balasse & Tresset, 2002; Tresset, 1996)); and meat (Bischoffheim, Early Neolithic, France (Gillis *et al.*, 2017)).

Finally, we estimate parameters for the later Neolithic and Early Bronze Age sites of Runnymede Bridge, Trasano IV-V, Font Juvenal, and Polgár Csőszhalom-dűlo (Gillis, 2012; Gillis *et al.*, 2016, 2017; Serjeantson, 2011) to examine if the cultural evolution (Richerson & Boyd, 2005) of slaughter strategies led to improvements in herd sustainability and the economic efficiency of calorie production over this period. Site phase details are provided in Supp. Table 3. These profiles were constructed using the method of Legge (1992) and Ducos (1968) (c.f. Bercy/Popina Bordusani).

### Sex-Specific Age Structure of Modern Unimproved Cattle Herds

We gathered data from 15 unimproved cattle herds that report the raw counts of male and female individuals at different ages (Amanor, 1995; Ducrotoy *et al.*, 2016; Ladan, 1992; Pullan, 1979; Trail *et al.*, 1980b; Wagenaar *et al.*, 1986; Wilson, 1986), see Supp. Table 1.

### Bayesian Model for Inferring Sexed Age-at-Death Profiles

We fit a Poisson regression with a random effect to the modern unimproved cattle data. Let ${t}_{j}$ be the middle point of age class $j, j=1,\dots , T$ and ${Y}_{sij}$ the number of surviving animals at time ${t}_{j}$ in herd $i$ for sex $s, s\in \left\{male, female\right\}$. Let $P{S}_{ij}$ denote the population size at time ${t}_{j}$ in herd $i$. We are interested in modelling the survival rate, *i.e.Y/*$PS$*.* To this end, we need to introduce an offset variable, which represents the size, exposure, measurement time, or population size of each observational unit. The regression coefficient for an offset variable is constrained to be 1, thus allowing the model to represent rates rather than counts. In the count regression model under consideration, the offset variable is equal to the log of the population size.

We assume that ${Y}_{sij}$ follow a Poisson distribution. Then, the Poisson regression model, including the offset, is given by:

$$E\left({Y}_{sij} \right)= {\mu }_{sij}$$

$$\mathrm{log}{\mu }_{sij} ={\eta }_{i}+\mathrm{log}P{S}_{ij}+{\beta }_{1}{t}_{j}+{\beta }_{2}{Sex}_{si}+{\beta }_{3}{Sex}_{si}\times {t}_{j}$$

where ${Sex}_{si}$ is an indicator variable for sex (1 = male and 0 = female). An interaction term between sex and time is included in the model to account for sex-specific differences in the effect of time. We specify independent flat prior distributions on the regression coefficients and assume that ${\beta }_{1}, {\beta }_{2}$, and ${\beta }_{3}$ are *a priori* independent and distributed according to a $N(\mathrm{0,10000})$, where $N\left(\mu ,{\sigma }^{2}\right)$ denotes the Gaussian distribution with mean $\mu$ and variance ${\sigma }^{2}$. As herd-specific random effect distribution, we assume ${\eta }_{i}\sim N\left(0,{\omega }^{2}\right),$ with $\omega \sim Uniform\left(\mathrm{0,100}\right).$ The model allows us to estimate temporal changes in the sex ratio while accounting for herd-specific heterogeneity. Crucially, it also enables inference for the ancient data, under the assumption that the sex ratio dynamics in ancient herds follow patterns similar to those observed in modern populations. This framework allows us to estimate the sex ratio in ancient herds by borrowing strength from contemporary data. We obtain:

$$r\left({t}_{j}^{\star }\right)=\mathrm{exp}\left\{{\beta }_{2}+ {\beta }_{3}{t}_{j}^{\star }\right\}$$

where ${t}_{j}^{\star }$ is the mid-point of age class $j$ of the ancient herd, $j=1,\dots ,{T}^{\star }$, and $r\left({t}_{j}^{\star }\right)$ is the sex ratio at time ${t}_{j}^{\star }$. In principle, more informative data on ancient herd sex ratio change (than modern unimproved herds) could be incorporated in the model, when available, such as kill-off profiles where sex had also been determined using, for example, ancient DNA methods.

Let ${N}_{kj}$ be the number of dead animals in ancient herd $k$ and $j$-th age-class, $k=1,\dots , M, j=1,\dots ,{T}^{\star }$. Note that the age class system does not need to be the same across modern and ancient herds. We assume that the vector of counts $\left({N}_{k1}, \dots , {N}_{k{T}^{\star }}^{\star }\right)$ has a multinomial distribution with size ${N}_{k\cdot }= \sum_{j=1 }^{{T}^{\star }}{N}_{kj}$ and vector of probabilities ${\theta }_{k}=\left({\theta }_{k1}, \dots , {\theta }_{k{T}^{\star }}\right)$, where ${\theta }_{kj}$ is the probability of dying in the $j$-th age-class for herd $k.$ We further impose a conjugate Dirichlet independent prior on the ${\theta }_{k}$, that is ${\theta }_{k}\sim Dirichlet\left(\gamma \right)$ where $\gamma =\left({\gamma }_{1},\dots ,{\gamma }_{T^\star }\right)$ indicates a ${T}^{\star }$-dimensional vector of strictly positive parameters. An advantage of our hierarchical formulation is that conjugacy can be exploited to integrate ${\theta }_{k}$ out, obtaining the Dirichlet–multinomial model,$\left({N}_{k1}, \dots , {N}_{k{T}^{\star }}^{\star } \right)\sim DM(\gamma )$, with probability mass function

$$p\left(n\left|\gamma\right.\right)=\frac{\Gamma\left(n_++1\right)\Gamma\left(\gamma_+\right)}{\Gamma\left(n_++\gamma_+\right)}\prod\nolimits_{j=1}^{T^\star}\frac{\Gamma\left({n_j+\gamma}_+\right)}{\Gamma\left(\gamma_j\right)\Gamma\left(n_j+1\right)}$$

with $n=\left({n}_{1},\dots , {n}_{{T}^{\star }}\right), {n}_{+}=\sum {n}_{j}, {\gamma }_{+}=\sum {\gamma }_{j}.$ The DM$\left(\gamma \right)$ allows more flexibility than the multinomial when encountering overdispersion in multivariate count data, as it induces an increase in the variance by a factor of $\left({n}_{+}+ {\gamma }_{+} \right)/\left(1+{\gamma }_{+}\right)$. We complete the model by specifying a prior distribution for $\gamma .$ More specifically, we assume

$${\gamma }_{j}\stackrel{iid}{\sim } TN\left(\mathrm{0,1000};0,\infty \right)$$

where $TN\left(\mu ,{\sigma }^{2};a,b\right)$ denotes a truncated normal distribution, truncated on the interval $(a,b)$. We are interested in estimating the proportion of males and females surviving per age class as well as the proportion of males killed due to sex ratio change combining information form modern and ancient data. Let ${f}_{k}=\left({f}_{k1},\dots , {f}_{kp}\right)$ and ${m}_{k}=\left({m}_{k1},\dots , {m}_{kp}\right)$ be the proportion of females surviving per age class and the proportion of surviving males, respectively, with $p={T}^{\star }-1$. In other words, ${m}_{kj}$ and ${f}_{kj}$ are the proportions of males and females still alive in age class *j*. In terms of standard life table notation, ${f}_{kj}$ (and similarly, ${m}_{kj}$) corresponds to ${l}_{x}$, (*i.e.* the percentage of the original cohort still alive at age *x*); ${\theta }_{kj}$ corresponds to ${q}_{x}$ (*i.e.* the probability of dying between ages *x* and *x* + 1), so that the probability of surviving, ${p}_{x}$, is equivalent to ${1-\theta }_{kj}$; finally, ${d}_{x}$ (*i.e.* the percentage of the cohort dying between ages *x* and *x* + 1) can be derived from ${N}_{kj}$. Under the above assumptions and definitions, the actual age-at-death profile can be rewritten as

$${\theta }_{kj}=\left[{m}_{kj-1}+{f}_{kj-1}\right]-\left[{m}_{kj}+{f}_{kj}\right]$$

with ${f}_{k0}={m}_{k0}=\frac{1}{2}, {f}_{k{T}^{\star }}= {m}_{k{T}^{\star }}=0$ and $\frac{{m}_{kj}}{{f}_{kj}}=r\left({t}_{j}^{\star }\right).$ Since ${\theta }_{kj}\ge 0$ and $\sum_{j=1 }^{T^\star }{\theta }_{kj}=1,$ we obtain ${f}_{kj }=\frac{1-{\theta }_{k1}-\cdots -{\theta }_{kj}}{1+r\left({t}_{j}^{\star }\right)}$, ${f}_{kj}\le {f}_{kj-1}$ and ${m}_{kj}\le {m}_{kj-1 }.$

We assume that the sex ratio at birth is 1:1. However, it should be noted that empirical estimates for the proportion of males at birth range from 51 to 53% male (Mendes *et al.*, 2022). Such a small range is unlikely to affect our main conclusions, but this uncertainty could be integrated into our model by specifying a suitable prior distribution.

Note that ${f}_{kj}-{f}_{kj-1}$ can be interpreted as the proportion of female animals that died in herd *k* in age class *j*. The term ${m}_{kj}-{m}_{kj-1}$ depends also on the proportion of males killed as part of the sex ratio change process. This latter proportion can also be estimated as $({m}_{kj-1}-{m}_{kj}){-(f}_{kj-1}-{f}_{kj })$.

To allow for uncertainty around the estimated sex ratio, we assume that ${f}_{kj}$ is distributed according to a Gaussian distribution with mean $\frac{1-{\theta }_{k1}-\cdots -{\theta }_{kj}}{1+r\left({t}_{j}^{\star }\right)}$ and variance 0.01, truncated on the interval (0, ${f}_{kj-1}$). Such a prior distribution corresponds to a vaguely informative prior as the standard deviation is equal to 0.1. We note that the values of ${f}_{kj}$ cannot be above 0.5 and are often much smaller (similarly for ${m}_{kj}$). If we compare with the variance of a uniform distribution on the interval [0, 0.5], which is approximately 0.02, we prefer a prior distribution more centred around the theoretical values while still allowing for uncertainty in the sex ratio.

An advantage of the Bayesian framework is that it can handle different sources of uncertainty in a probabilistically sound way. In our context, some age-at-death profiles include uncertain age class assignment (*i.e.* individual animals may be attributed to multiple adjacent age classes). Assuming *a priori* equal assignment probabilities to each age class, we can impute the assignment of each individual animal from the appropriate conditional posterior distribution at each iteration of the MCMC algorithm.

We ran two independent chains of the MCMC algorithm, each for 110,000 iterations with the first 10,000 discarded as burn-in, and kept every 10th iteration (thinning) yielding 20,000 samples from the posterior distribution. Supp. Figure 1 shows good mixing for a subset of the parameters. Gelman and Rubin’s shrink factor (Gelman & Rubin, 1992), a formal test for convergence presented in Supp. Figure 2 and computed on 4 independent runs, shows a shrink factor close to 1 after 5000 iterations. This indicates that the total number of iterations and the burn-in are large enough for the chain to quickly converge to the target density, also corroborated by stable rolling means and 2.5th and 97.5th quantiles shown in Supp. Figure 3. We see little autocorrelation between successive values in the chains in Supp. Figure 4, meaning that the level of thinning is sufficient.

Even though we tuned the MCMC algorithm to work well on commonly expected input sizes, ultimately no set of MCMC parameters (*i.e.* number of iterations, burn-in, thinning, and proposal function window sizes) fits all possible inputs, and sanity checking the mixing of the chains and potentially adjusting the parameters is common practice when using any MCMC.

### Methodology Validation

**Fig. 1:**
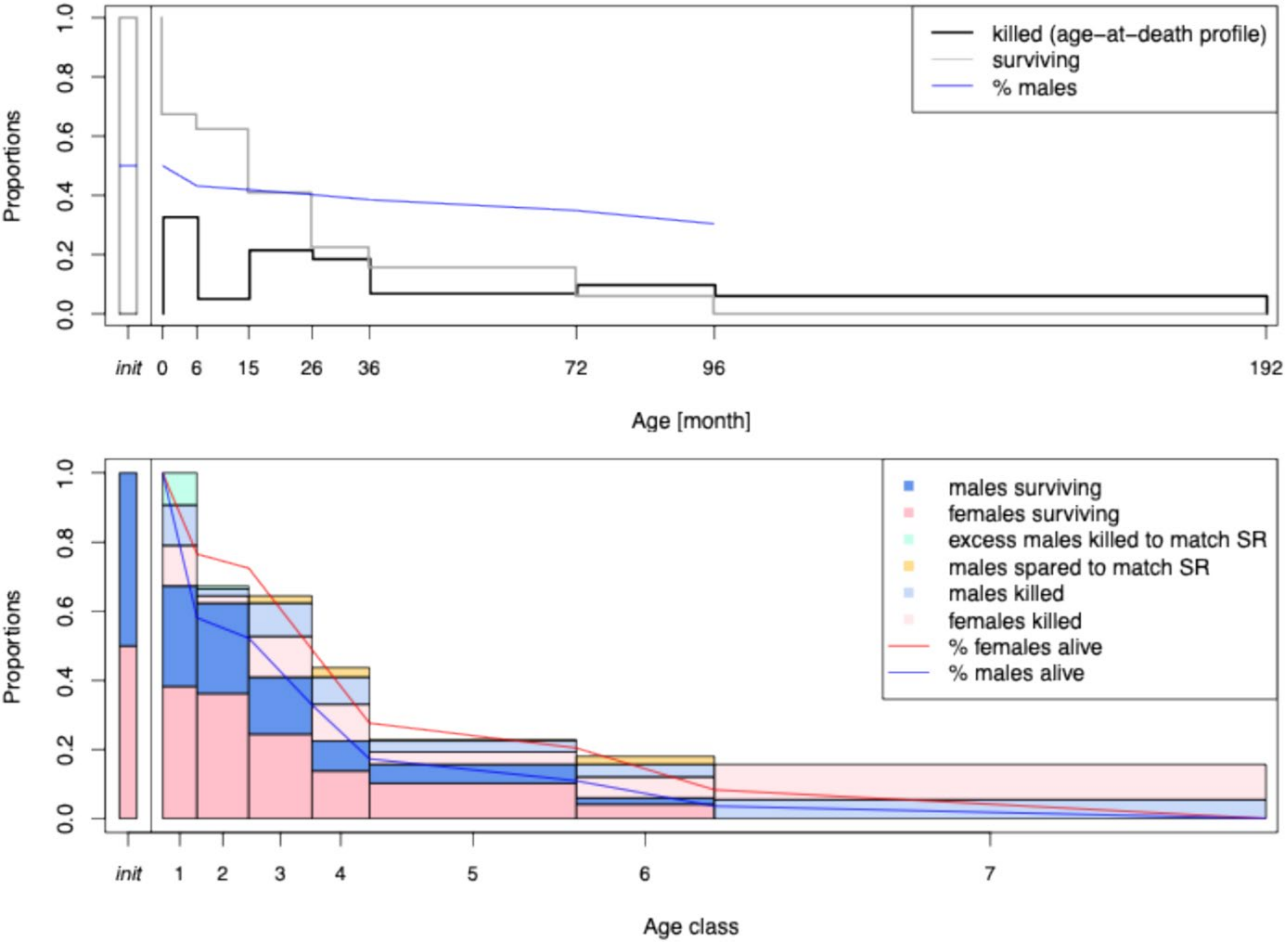
The upper panel shows a toy illustration of the model. The black line shows the actual kill off profile; the grey line represents the survival curve for the population, and the blue line represents the proportion of surviving males, assuming a sex ratio of 1:1 at birth. The lower panel details the inferred values for the model parameters

A straightforward way to validate the model and inference scheme described in the previous sections is to simulate data under the model with known parameter values and compare those to the values inferred by MCMC. Figure 1 displays a toy illustration of the model.

To assess the accuracy of our method across the different age classes and given different number of individuals, we adopt a procedure similar to Bayesian *p*-values, which are usually implemented as a measure of goodness-of-fit. From the posterior predictive distribution of *f*,* m*, and *r*, we sampled 50 values for a hypothetical ancient herd, and from this, we derive values for *θ*. We sample draws from a multinomial distribution with parameter *θ* and size $n\in \left\{10, 20, 50, 75, 100, 150, 200\right\}$. This generated a total of 350 different simulated age-at-death profiles with known underlying parameter values.

### Estimation of Animal Productivity Parameters

In order to infer meat and offal production from slaughtered animals, it is necessary to have a model of animal weight by age. We used the model presented in Vigne (1991) for weight $\nu$ in month $x$

$$\nu = \tau \times {e}^{\frac{a}{b}\left(1-{e}^{-bx}\right)}$$

where $\tau$ is the birth weight, and parameters $a$ and $b$ determine the rate and shape of the growth curve. We fitted values for parameters $\tau , a,\text{ and }b$ by least squares using a search method and constraining the value of the parameters in the intervals: $\tau \in$ [10, 30], $a\in$(0, 1], $b\in$ (0, 1] (see Supp. Figure 5), using published weight data (see Supp. Table 2) from unimproved African herds (Hoste *et al.*, 1992; Otchere, 1986; Trail *et al.*, 1980a, 1980b; Wagenaar *et al.*, 1986; Wilson, 1986).

To estimate archaeological animal productivity parameters, for each MCMC sample, we took the probability of survival by sex by age for each month and multiplied these by ethnographic estimates of mean milk produced (Hoste *et al.*, 1992; Otchere, 1986; Trail *et al.*, 1980b; Wagenaar *et al.*, 1986), milk consumed by calves (Hoste *et al.*, 1992; Otchere, 1986; Wagenaar *et al.*, 1986), meat and offal weight (MOW) produced (Trail *et al.*, 1980a, 1980b), and probability of giving birth (Hoste *et al.*, 1992; Ladan, 1992; Otchere, 1986; Trail *et al.*, 1980a; Wagenaar *et al.*, 1986), for each month of life in unimproved African herds. For milk yield, we averaged estimates from four studies (Hoste *et al.*, 1992; Otchere, 1986; Trail *et al.*, 1980b; Wagenaar *et al.*, 1986), yielding a value of 42.3 kg per month when calves were suckling, and assumed that milk production began at 42 months (based on average age at first calving, see Hoste *et al.*, 1992; Ladan, 1992; Otchere, 1986; Trail *et al.*, 1980a; Wagenaar *et al.*, 1986). Given an average calving interval of 18.36 months, and an average suckling period of 9.18 months (Hoste *et al.*, 1992; Ladan, 1992; Otchere, 1986; Trail *et al.*, 1980a; Wagenaar *et al.*, 1986), this gives an average amount of milk produced per cow per month of 21.13 kg. Based on three studies, we estimated the proportion of milk consumed by calves as 68.4% (Hoste *et al.*, 1992; Otchere, 1986; Wagenaar *et al.*, 1986); this was subtracted from milk produced to give milk yields available for human consumption of 6.68 kg per month. From the above estimated calving interval, we calculated the probability of giving birth (post 42 months) as 0.0545 per month. We estimated that MOW yield was 49.95% of live weight (Trail *et al.*, 1980a, 1980b). For macronutrient and energy yields (protein, fat, and calories) from milk and MOW, we used data from the US Department of Agriculture National Nutrient Database for Standard Reference (Haytowitz, 2015). This indicated that milk produces 0.0333 kg of protein, 0.0375 kg of fat, and 670 kcal per kg, and meat and offal produce 0.1942 kg of protein, 0.1273 kg of fat, and 1980 kcal per kg. By summing across all months of life, multiplied by the probabilities of animals being alive at those ages, average yields per animal per lifetime were obtained.

Since we derive calories obtained from milk and MOW per month for female and male animals at various ages, it is desirable to estimate the cost of producing those calories (*i.e.* the costs of keeping animals). With such estimates, it would be possible to approximate the relative economic efficiency of calorie production for various slaughter strategies. There are no obvious units for such costs in a prehistoric context, but a first-order-of-approximation proxy should be the weight of the animals themselves (*i.e.* larger animals require more resources, such as labour, feed, and land, to keep). For convenience, we express this cost as the estimated feed consumed per month at different ages. Dikshit and Birthal () estimate an average daily consumption of dry matter as 2.475% of live weight in Indian cattle. This translates to 75.33% of live weight per month, which we use as a general proxy for the economic cost of keeping animals. We then divide estimated calorie yields per month by these estimated economic costs of keeping animals to approximate the economic efficiency of calorie production for different slaughter strategies. It is important to note that we only use these values as a relative measure of economic costs and efficiencies in order to compare different kill-off profiles.

To find kill-off profiles that maximise the possible yields of key derived products (MOW, milk, economic efficiency of calorie production, reproductive output rates), given the assumed per individual per age ethnographic consumption and yield parameter estimates from unimproved stock used here, we simulated kill-off profiles under two scenarios, with 20,000 simulations for each:

1. Profiles simulated under our model, as described in the ‘Methodology Validation’ section above (sex-asymmetric strategies).
2. Profiles simulated by sampling $\psi$ from a ${\mathrm{Dir}}\left({\alpha }_{i}=1\right)$ and then setting female and male proportions of death in each age class equal to $\frac{\psi }{2}$ (sex-symmetric strategies).

## Results

### Methodology Validation

**Fig. 2:**
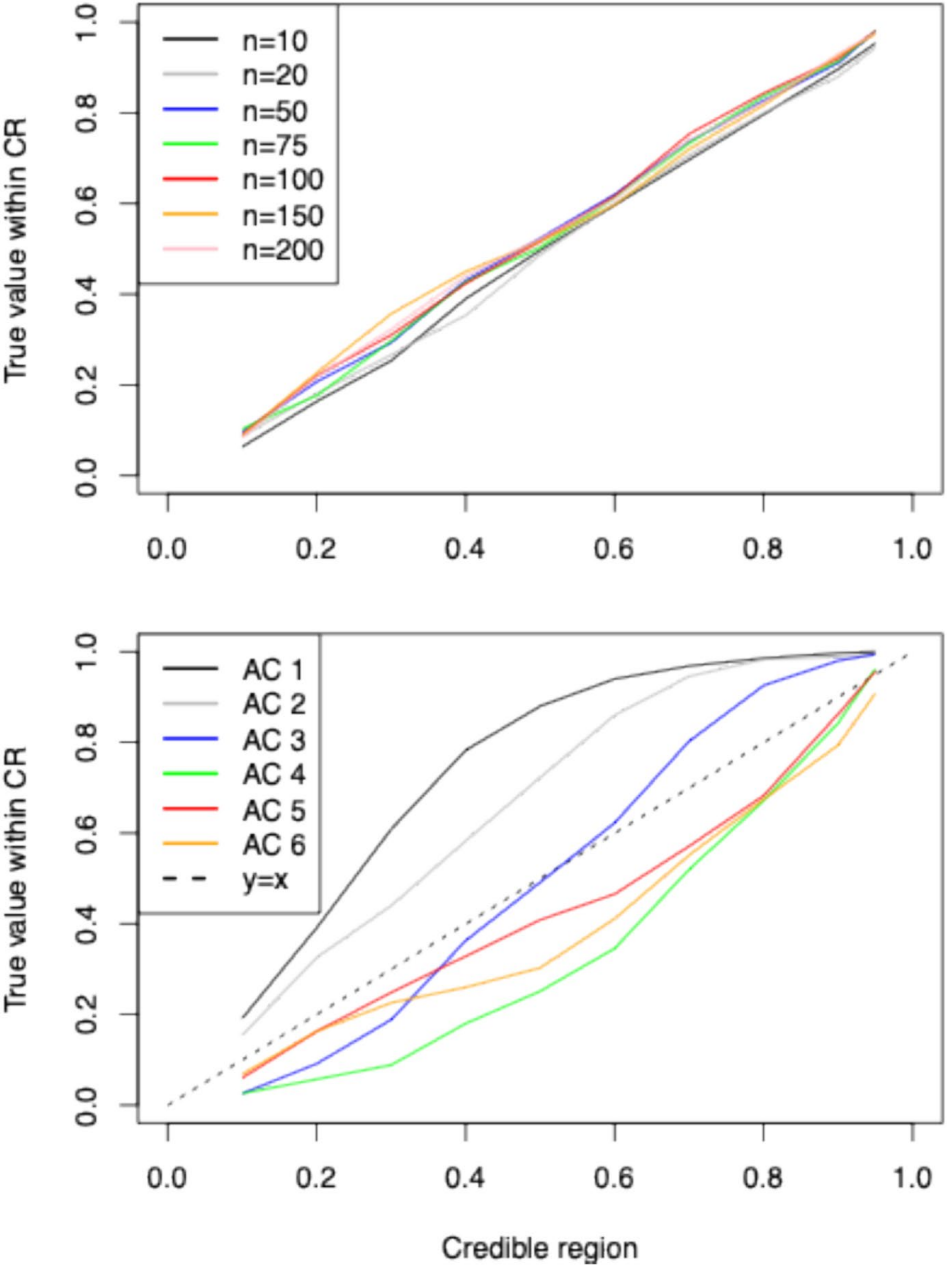
Accuracy of inferential approach based on 350 simulated datasets described in the ‘Materials and Methods’ section. Accuracy is measured as the proportion of simulated datasets for which the 95%, 90%, 80%, 70%, 60%, 50%, 40%, 30%, 20%, and 10% highest posterior density (HPD) interval obtained from the posterior distribution of ***f*** contains the true simulated parameter value for each age class. In the upper panel, we show the results for different sample sizes, and in the lower panel, we show the results for different age classes

Figure 2 summarises the results of our validation on the 350 simulated datasets described in the ‘Materials and Methods’ section. We measure the accuracy of the proposed inference scheme by reporting the proportion of simulated datasets for which the 95%, 90%, 80%, 70%, 60%, 50%, 40%, 30%, 20%, and 10% highest posterior density (HPD) interval obtained from the posterior distribution of ***f*** contains the true simulated parameter value for each age class. Our results show that our model recovers the true age-at-death profile with good accuracy. Interestingly, the upper panel of Fig. 2 shows that accuracy is high even for a low number of individuals. This is encouraging as we expect typical archaeological datasets to be small. The lower panel of Fig. 2 shows slight differences in the accuracy across age classes: The model performs best for the younger age classes, which is to be expected since these age classes contain more observations.

### Yield and Growth Estimates from Idealised Kill-Off Profiles

McGrory *et al.* (2012) suggested an idealised cattle kill-off profile that was optimized for milk production. Since ‘idealised’ kill-off profiles are—by definition—not empirical, they must be expressed as proportions of animals killed in each age class. Conditionally on these proportions, we can simulate from the predictive distribution of ***f*** and ***m*** integrating also information on sex ratio, as described in the ‘Materials and Methods’. In what follows, we compare results from empirical datasets with those obtained from the idealized profile.

In addition to McGrory *et al.*’s (2012) ‘idealised’ cattle kill-off profile targeting milk production, Legge (1992) hypothesised that an archaeological kill-off profile from the UK Bronze Age site of Grimes Graves represents a management strategy targeting intensive milk production, involving the slaughter of male calves at birth. In contrast, the kill-off profiles from Popina Bordusani (Eneolithic, Romania) and Bercy (Middle Neolithic, France) were interpreted (based on stable isotope and mortality data) as representing management strategies that targeted slaughtering male calves post-lactation and were hypothesised to be associated with mixed dairy and meat production. Finally, we included Bischoffsheim (Early Neolithic, France), which had been proposed to be a profile representing meat-focused strategies (Gillis *et al.*, 2017). This interpretation was supported by low frequency of milk lipids found in ceramic sherds (Casanova *et al.*, 2020). For convenience, we refer to these five kill-off profiles as ‘idealised’, even though four of them are based on empirical data.

**Fig. 3:**
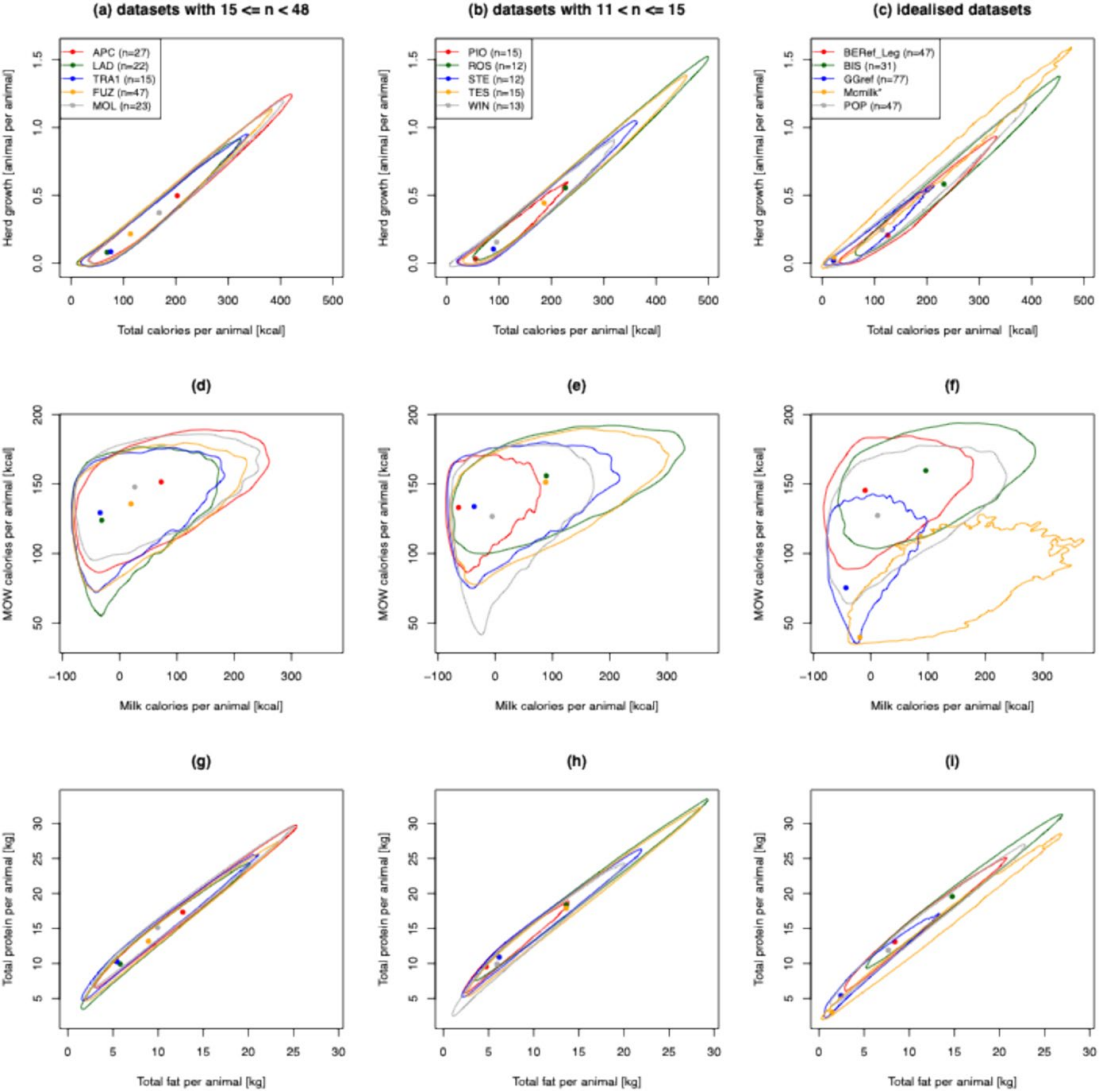
Joint posterior estimates of herd growth (animals per animal) versus total calories per animal (**a)**–(**c**), MOW calories per animal versus milk calories per animal (**d)**–(**f**), and total protein per animal versus total fat per animal (**g)**–(**i**) for different archaeological sites and idealized slaughter strategies. Idealised strategies profiles (**c)**, (**f)**, (**i**) are Popina Bordusani (POP); Bercy (BEref); Bischoffheim (BIS), McGrory hypothetical milk profile (Mcmilk) and Grimes Grave (GGref). Largest (**a)**, (**d)**, (**g**) and smallest (**b)**, **(e)**, (**h**) refer to the five archaeological sites with the largest (Apc-Berekalja, APC; La Draga, LAD; Trasano I, TRA1; Füzseabony-Gubakút, FUZ; and Mold, MOL) and five sites with the smallest number of individuals (Polgar-Piócási-dűlo, PIO; Rosheim, ROS; Stephansposching, STE; Těšetice-Kyjovice, TES; and Windmill Hill, WIN)

**Fig. 4:**
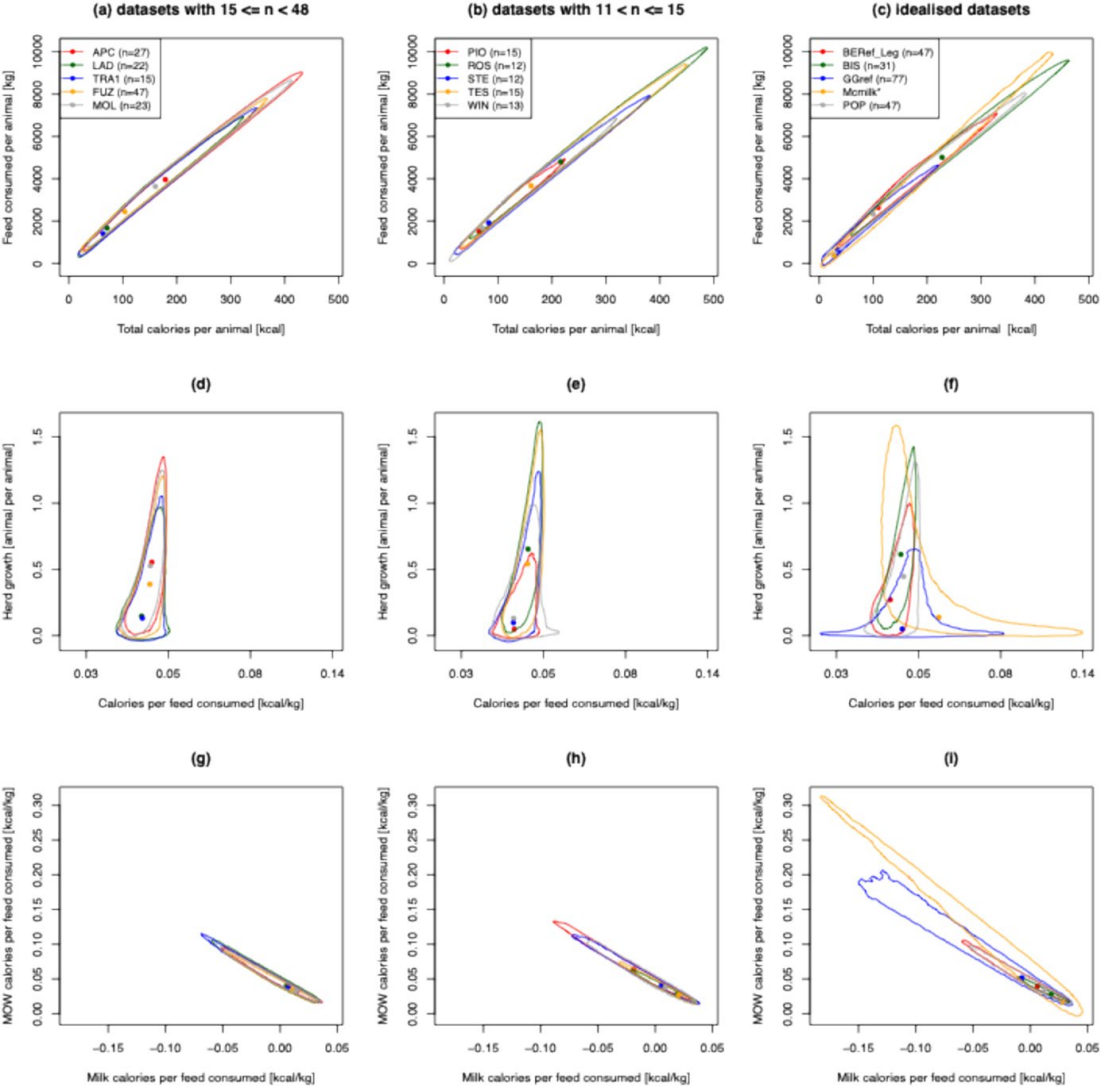
Joint posterior estimates of feed consumed per animal (kg) versus total calories per animal (**a)**–(**c**), herd growth (animals per animal) versus calories per feed consumed (kcal/kg; (**d)**–(**f)**), and MOW calories per feed consumed (kcal/kg) versus milk calories per feed consumed (kcal/kg; (**g)**–(**i)**) for different archaeological sites and idealized slaughter strategies. Idealised strategies profiles (**c)**, (**f)**, (**i**) are Popina Bordusani (POP), Bercy (BEref), Bischoffheim (BIS), McGrory hypothetical milk profile (Mcmilk), and Grimes Grave (GGref). Largest (**a)**, (**d)**, (**g**) and smallest (**b)**, (**e)**, (**h**) refer to the five archaeological sites with the largest (Apc-Berekalja, APC; La Draga, LAD; Trasano I, TRA1; Füzseabony-Gubakút, FUZ; and Mold, MOL) and five sites with the smallest number of individuals (Polgar-Piócási-dűlo, PIO; Rosheim, ROS; Stephansposching, STE; Těšetice-Kyjovice, TES; and Windmill Hill, WIN)

For these ‘idealised’ profiles, we examined their inferred milk and MOW calorie yields per animal (Fig. 3f), milk and MOW calorie yields per feed consumed (Fig. 4i), total calorie yields (milk + MOW; Fig. 4f), and predicted reproductive output rates (defined as animals produced per animal) assuming a birth rate of 1 calf every 18.36 months (Hoste *et al.*, 1992; Ladan, 1992; Otchere, 1986; Trail *et al.*, 1980a; Wagenaar *et al.*, 1986) (Figs. 3c and 4f). We note that McGrory *et al.*’s () idealised kill-off profile targeting milk production is predicted by our approach to give low milk calories per animal and the lowest MOW calories per animal (among all kill-off profiles considered in this study), but the highest milk calories per feed consumed and the lowest MOW calories per feed consumed. Thus, from an economic standpoint, this idealised kill-off profile can be seen as targeting milk over MOW production. However, our approach predicts the kill-off profile from Grimes Graves to yield the lowest milk and highest MOW calories per feed consumed, suggesting it should be seen as a meat production kill-off strategy. Finally, while the Bischoffsheim kill-off profile is predicted to yield the highest calories per animal, including both the highest milk calories per animal, and MOW calories per animal (among all kill-off profiles considered in this study), it is also predicted to yield intermediate MOW and milk per feed consumed (again, among all kill-off profiles considered in this study). It is notable that our approach also predicts that the Bischoffsheim kill-off profile would yield the highest herd growth rate. These predictions result mainly from an excess of older animals at the site.

### Analysis of Neolithic Archaeological Kill-Off Profiles

As can be seen from Figs. 3 and 4, all output parameter 95% credible regions are larger for the five sites with the smallest sample sizes than they are for the five sites with the largest sample sizes. This reflects uncertainty in the underlying age-at-death profile due to sampling. However, it is notable that for most output parameters, the 95% credible regions are overlapping, suggesting caution should be taken when comparing product yield estimates across sites.

By comparing the modes of the product yields estimates (*i.e.* point estimates) across the five sites with the largest sample sizes, we infer that they have similar economic efficiencies of calorie production (Fig. 4d). It is notable that these five sites were previously considered similar in terms of slaughter management strategies (Gillis *et al.*, 2017). However, it is noted that Apc-Berekalja and Mold had a greater number of teeth from adult animals, which is consistent with their higher inferred herd growth rates.

Our model, when combined with the ethnographic data from modern unimproved African cattle, suggests a broad negative relationship between MOW calories per feed consumed and milk calories per feed consumed (Fig. 4g–i). Thus, as expected, milk and MOW calorie production appear traded off against one another. For all ten Neolithic sites and four of the five model kill-off profiles (*i.e.* not Mcmilk), we infer higher MOW than milk calories per feed consumed (Fig. 4g–i). We note that both Ingold (1980) and Legge (1981, 1992) have suggested that milk production was more economically efficient than meat production. Our results seemingly contradict this view, suggesting either that the economic efficiency of meat calorie production has been underestimated or more likely that the ethnographic parameters we obtained from modern unimproved African cattle (Dikshit & Birthal, 2010; Hoste *et al.*, 1992; Ladan, 1992; Otchere, 1986; Trail *et al.*, 1980a, 1980b; Haytowitz, 2015; Wagenaar *et al.*, 1986) are not appropriate for inferring Eurasian Neolithic cattle calorie yields (but see below). Consistent with this, we note that the ethnographic milk yield parameter estimates from unimproved African cattle used here are typically lower than those for unimproved Eurasian cattle (Gillis, 2017), possibly reflecting the high levels of aridity in many of the regions where the African ethnographic data was gathered. Indeed, milk yield estimates for modern unimproved Turkish cattle (Soysal & Kök, 2006; Soysal *et al.*, 2003) are more than fourfold higher.

Another indicator that the ethnographic parameters we obtained from modern unimproved African cattle (Dikshit & Birthal, 2010; Hoste *et al.*, 1992; Ladan, 1992; Otchere, 1986; Trail *et al.*, 1980a, 1980b; Haytowitz, 2015; Wagenaar *et al.*, 1986) are most likely not appropriate for inferring Eurasian Neolithic cattle outputs is that our point estimates of herd growth rates are all below one (Figs. 3 and 4), suggesting non-sustainable herds. We note that the values we obtained from African cattle for age at first calving (42 months) and average calving interval (18.36 months)—the two parameters most likely to affect herd growth rates (alongside the kill-off profile)—are higher than those of modern Holstein cattle (24 months and 13.27 months, respectively (Atashi *et al.*, 2021)), although lower than those estimated for modern unimproved Turkish cattle (Soysal & Kök, 2006; Soysal *et al.*, 2003). To better understand the role of these parameters, we repeated the analyses shown in Fig. 4d–f, using 24 months and 13.27 months for age at first calving and average calving interval, respectively (see Supp. Figure 6). This brings all herd growth point estimates closer to or above 1, and all 95% CIs on this output to include 1.

### Maximising Yields

From an economic sustainability point of view, total calories (milk and MOW) produced per feed consumed and reproductive output rates are likely the two most valuable targets in any slaughter strategy; the former to maximize the economic efficiency of nutritional return and the latter to maximize herd resilience. We note that when assuming the ethnographic yield parameter values from modern unimproved African cattle (Dikshit & Birthal, 2010; Hoste *et al.*, 1992; Ladan, 1992; Otchere, 1986; Trail *et al.*, 1980a, 1980b; Haytowitz, 2015; Wagenaar *et al.*, 1986), there appears to be scope for improving herd growth without sacrificing the economic efficiency of calorie production (Fig. 4d–f). Our approach allows us to explore the full range of possible slaughter strategies in order to identify those that maximize total calories (milk and MOW) produced per feed consumed and/or reproductive output rates. Identifying such theoretical slaughter strategies will sharpen our intuitions on the scope for improvement of the slaughter strategies represented in archaeological kill-off profiles.

**Fig. 5:**
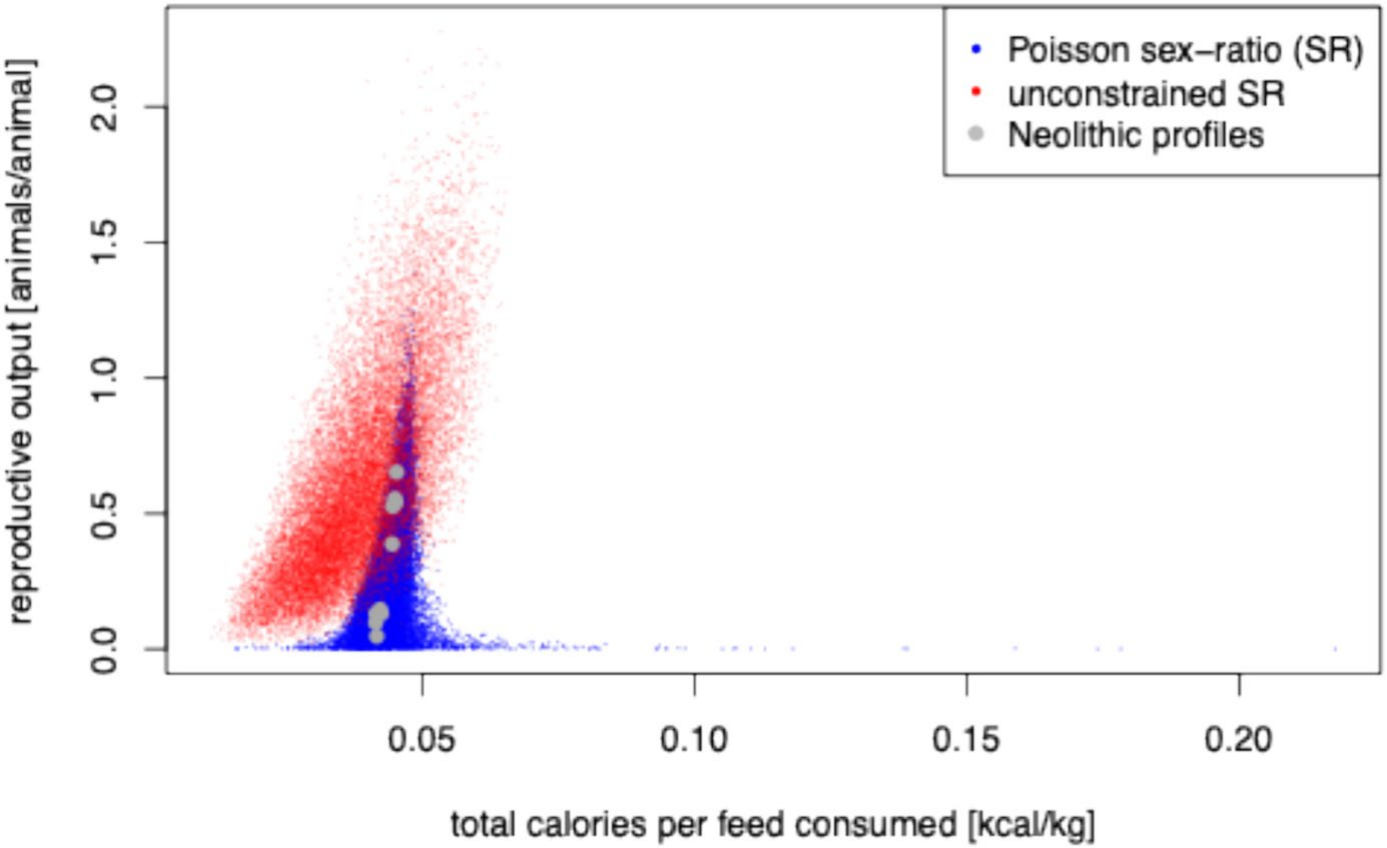
Estimates of herd growth (animals per animal) versus calories per feed consumed (kcal/kg) for 20,000 kill-off profiles simulated under two different scenarios: (1) the sex-asymmetric model presented here (blue dots) and (2) a sex-symmetric model where the total number of animals killed in each age class is unconstrained with males and females killed in the same proportions (red dots). In addition, the posterior mode values inferred from the empirical kill-off profiles analysed in Fig. 4d–f are shown (large grey dots)

To investigate this further, we consider two simulation scenarios: one involves sampling data from the model presented here (sex-asymmetric strategies), and one which assumes that the total number of animals killed in each age class is unconstrained with males and females killed in the same proportions (sex-symmetric strategies; see ‘Materials and Methods’). We generated 100,000 random kill-off profiles from each (see ‘Materials and Methods’). In both cases, we identified kill-off strategies where the economic efficiencies of calorie production and reproductive output rates were higher than both those of the ten Neolithic archaeological kill-off profiles and the five idealised profiles considered here (Fig. 5). However, we note that while the ‘sex-symmetric’ search identified slaughter strategies that perform better when considering both calorie production and herd security—as expected given that it explores a larger parameter space—the ‘sex-asymmetric’ search leads to the identification of strategies giving relatively high values for both outcomes, including ones that are higher than inferred for any of the ten Neolithic age-at-death profiles analysed here. From Fig. 5, we note that there is a long tail along the *x*-axis, representing theoretical kill-off profiles giving very high values for the economic efficiency of calorie production. However, these profiles are unrealistic; they involve killing animals of both sexes at a very young age, leading to near-zero reproductive outputs. Because herd growth rates below 1 are, by definition, unsustainable, culturally transmitted kill-off strategies leading to herd growth rates < 1 are likely to be short-lived. The theoretical kill-off profile giving the highest economic efficiency of calorie production, assuming a sustainable herd (*i.e.* conditionally on herd growth > 1; see Supp. Figure 7) involves killing most males between 0 and 26 months (according to the Legge age class schema (Legge, 1992)). Indeed, this theoretical kill-off profile is predicted (assuming the ethnographic parameters we obtained from modern unimproved African cattle) to yield around 110 to 120% of the economic efficiency of calorie production as any of the ten Neolithic sites, and at least around twice the reproductive output (Fig. 5). The theoretical kill-off profile giving the highest herd security unsurprisingly involves allowing females to live until their maximum age.

Interestingly—even when using the ethnographic yield parameter values from modern unimproved African cattle (Dikshit & Birthal, 2010; Hoste *et al.*, 1992; Ladan, 1992; Otchere, 1986; Trail *et al.*, 1980a, 1980b; Haytowitz, 2015; Wagenaar *et al.*, 1986)—we find that most herd-sustainable (*i.e.* herd growth > 1) kill-off strategies lead to higher milk than MOW calories per feed consumed (see Supp. Figure 8). This is consistent with the claims of Ingold (1980) and Legge (1981) that milk production was more economically efficient than meat production.

### Analysis of Later Neolithic and Post-Neolithic Kill-Off Profiles

Our exploration of theoretical kill-off profiles indicates that there was scope for improvement of the Neolithic kill-off strategies analysed above. Under a general model of cultural evolution (Richerson & Boyd, 2005), we would therefore expect some improvement in the predicted economic efficiency of calorie production and perhaps reproductive output rate, with time. To examine this, we performed our analysis on four age-at-death profiles from archaeological sites dating from later Neolithic periods (*i.e.* from the 4th millennium BC). As can be seen from Supp. Figure 9, while there is overlap in estimates, there is no obvious trend of increasing reproductive output rates, nor increasing calories per feed consumed, at the later sites. However, it is important to note that (1) the small number of sites considered here only span the Neolithic, and (2) this analysis was performed using the ethnographic yield parameter estimates from unimproved African cattle (Dikshit & Birthal, 2010; Hoste *et al.*, 1992; Ladan, 1992; Otchere, 1986; Trail *et al.*, 1980a, 1980b; Haytowitz, 2015; Wagenaar *et al.*, 1986), which may not be appropriate for prehistoric European cattle.

## Discussion

We present a Bayesian method and associated MCMC algorithm for inferring sex-specific survival curves from unsexed cattle archaeological age-at-death profiles. Our approach exploits modern ethnographic data to inform a survival model for prehistoric data, providing information on sex-ratio change by age. Data-driven estimation of sex-ratio change is performed assuming a Poisson model for counts of surviving animals in unimproved herds. In a Bayesian framework, such information is easily transferred in a probabilistically coherent way to model sex-specific survival in prehistoric herds, for which only unsexed death count data are available. A major advantage of the Bayesian framework is the ability to deal with uncertainty in assignment to individual age class, as well as not being restricted to a particular age class schema (Ducos, 1968; Legge, 1992; O'Connell, 1991). As with all models, some of the assumptions made in this work are simplistic, in particular assuming that sex ratio change by age is similar in prehistoric and modern herds. One of the challenges in working with prehistoric datasets is that data are often incomplete, inaccurate, and biased. We contend that integrating information from modern herds can offer valuable insights, and our model is able to capture important aspects of slaughter management and, as such, is useful (Box, 1976). Indeed, our approach permits the identification of slaughter strategies with both high economic efficiencies of calorie production and high reproductive output rates (see Fig. 5). Whilst it is highly unlikely that Neolithic herders understood optimal models of sex ratio change by age, the idea of killing males early could have been associated with ritual beliefs as a proximate cause, with the ultimate outcome being the cultural evolution of economically improved herd management strategies (Mayr, 1961; Richerson & Boyd, 2005).

It is important to note that the estimates of prehistoric herd productivity presented here are dependent on the ethnographic productivity data at different ages and sexes from modern unimproved African cattle. Our approach should be seen as a means of combining prehistoric kill-off data with ethnographic slaughter management and productivity data, not as a means of inferring prehistoric herd productivity from kill-off data alone. Indeed, we note that there are reasons to question the suitability of the African ethnographic productivity data for prehistoric European cattle: (1) The modes of the inferred herd growth rates made using the Neolithic kill-off profiles considered here (Gillis *et al.*, 2017) are all below 1, some considerably so; (2) in contrast to suggestions of Ingold (1980) and Legge (1981) that milk production was more economically important than meat production, we infer higher MOW than milk calories per feed consumed for the ten Neolithic kill-off profiles considered here; (3) the African productivity data was obtained from cattle living in mostly arid regions, whereas Neolithic Europe would have been more temperate with ample precipitation; and (4) Gregg (1988) estimated that the milk available for human consumption in Neolithic Europe was also almost twice that used in this work. It is notable that when we assume the age at first calving and average calving interval—the two parameters most likely to affect herd growth rates—are the same as those of modern Holstein cattle, our herd growth point estimates for Neolithic cattle are closer to or above 1, and all 95% credible regions on this output include 1 (see Supp. Figure 6).

In our analyses, we have allowed herd growth to vary freely. However, it is reasonable to assume that—to a first-order-of-approximation—most prehistoric herd management strategies were sustainable and allowed limited herd growth. If this were the case, then we could modify our model by constraining the parameters to accommodate only herd growth rates greater than one. Indeed, by conditioning on kill-off strategies ensuring herd growth above 1 in our exploration of theoretical ‘sex-asymmetric’ kill-off profiles (Supp. Figure 8), we find that most random strategies lead to higher milk than MOW calories per feed consumed, in agreement with the claims of Ingold (Ingold, 1980) and Legge (Legge, 1981) that milk production was more economically efficient than meat production.

It should also be noted that our estimates of feed consumed in a prehistoric context are not intended to represent the total food consumed by each animal. Rather, we use them as a general proxy of the economic costs of keeping animals at different ages. For this reason, our estimates of product yield per feed consumed should only be considered in relative terms, rather than as an absolute measure of product yields per feed consumed. We also recognize that prehistoric values for such parameters may have been different, in most cases likely lower. However, the methodology we have developed is not tied to the particular values of the parameters used here, and other values—if better informed—could be used. Nonetheless, predicted yields per animal from our model scale linearly with these parameter values, so while our absolute yield estimates may be misleading, their relative values in cross-site and site/model comparisons should be reasonably reliable.

By exploring the space of slaughter strategies, we identified theoretical kill-off profiles that should lead to higher herd growth rates and calorie yields per feed consumed than those inferred for the archaeological data considered here (Fig. 5). Given the importance of these target outcomes, it therefore seems likely that there was scope for improvement of Neolithic slaughter strategies through cultural evolutionary processes (Richerson & Boyd, 2005). However, we did not infer any such improvement when considering later Neolithic data. This may be because the sample sizes and temporal range considered here were quite small, and/or because the ethnographic yield parameter estimates from unimproved African cattle (Dikshit & Birthal, 2010; Hoste *et al.*, 1992; Ladan, 1992; Otchere, 1986; Trail *et al.*, 1980a, 1980b; Haytowitz, 2015; Wagenaar *et al.*, 1986) are not appropriate for prehistoric European cattle. Alternatively, the prehistoric kill-off profiles considered here might result from strategies adapted to different ecological zones or the seasonality of site usage. It should be noted that the theoretical kill-off profile giving the highest combination of economic efficiency of calorie production and reproductive output rate (Supp. Figure 7) involves allowing all females to live to their maximum age. This is unrealistic as there will always be some female mortality prior to maximum age, due to predation, disease, starvation, communal feasting, or ritualistic slaughter (*e.g.* Windmill Hill, a causeway enclosure), as well as loss of fertility. Given a sufficient number of sites with informative age-at-death data, it should be possible to apply the methodology described here in a meta-analysis to test if there was any significant improvement over a wider span of time in herd sustainability or the economic efficiency of calorie production.

## Supplementary Information

Below is the link to the electronic supplementary material.

ESM 1 (XLSX 59.6 KB)

ESM 2 (DOCX 1.53 MB)

## Data Availability

All data used in this paper are provided in tables or from cited publications.

## Declarations

### Significance Statement

Archaeologists have used age-at-death estimates of prehistoric domestic animals to infer whether herds were kept primarily for meat, milk, or other secondary products, such as fleece. In principle, age-at-death profiles, in combination with information on the sex of individuals, would allow predictions of milk and meat production, as well as herd reproductive rates and the economic efficiency of calorie production for different slaughter strategies. We present a Bayesian approach for inferring the economic efficiency of milk, meat, calorie production, and herd growth rates from unsexed age-at-death profiles, in combination with ethnographic data on age and sex from modern unimproved cattle herds. We find that there was scope for improvement in slaughter management.

### Computer Code

All MCMC code was written in R and JAGS. All plotting functions and ancillary code were written in R. Code is available via the following GitHub repository: https://github.com/emptyjasus01/AsymetricSexRatioModel/.
