# Supplementary material for: Bayesian Inference of Sex-Specific Mortality Profiles and Product Yields from Unsexed Cattle Zooarchaeological Remains
Source: J Archaeol Method Theory. 2025 Nov 6;33(1):13. doi: 10.1007/s10816-025-09749-x (PMC12592301; doi:10.1007/s10816-025-09749-x)
Supplement: Supplementary file 2 — (DOCX 1.53 MB) [file 10816_2025_9749_MOESM2_ESM.docx]

**Supporting Information**

For Supporting Tables, see separate file

**Supp Table Legends:**

**Supp. Table 1**

Sex ratio change data obtained from modern unimproved cattle herds (Pullan 1979; Trail *et al.* 1980; Wagenaar *et al.* 1986; Wilson 1986; Services 1992; Amanor 1995; Ducrotoy *et al.* 2016) and used to inform on the Poisson regression model.

**Supp. Table 2**

Published animal weight by age data from unimproved African herds (Trail *et al.* 1979; Trail *et al.* 1980; Otchere 1986; Wagenaar *et al.* 1986; Wilson 1986; Hoste *et al.* 1992). This data was used to estimate parameters of the model presented in Vigne (Vigne 1991), which in turn were used to estimate animal weights at different ages in months.

**Supp. Table 3**

Cattle age-at-death data (after Legge 1992; Ducos 1968) from early farming sites from UK, Italy, France, Austria, Hungary, Germany and Czech Republic. Hypothetical milk profile from McGrory et al. (2012) based on modern dairy cattle. EN: Early Neolithic; MN: Middle Neolithic; LBK:  Linearbandkeramik ; TLP:  Transdanubia pottery ALP: Alföld Linear Pottery.

**Supporting figures**

**Supp. Fig. 1**

MCMC trace plot visualising chain mixing (see Material and Methods for details).

**Supp. Fig. 2**

Gelman and Rubin’s shrink factor test for MCMC convergence on 4 independent chains (see Material and Methods for details).

**Supp. Fig. 3**

Rolling means and 2.5th and 97.5th quantiles of MCMC chain to assess convergence.

**Supp. Fig. 4**

Autocorrelation between successive values in the chain to assess if thinning is intervals are sufficiently wide.

**Supp. Fig. 5**

Estimated animal weight growth curve, following the model of Vigne (Vigne 1991), with parameter values obtained by least squares based on weight data from unimproved African herds shown as dots (see Materials and Methods for details).

**Supp. Fig. 6**

Results shown in Figure 4d-f re-computed with modern birth interval (13.27 months) and age at first birth (24 months). Abbreviations: birth rate (br), age at first birth (afb), month (m).

**Supp. Fig. 7**

Simulated profile maximising economic efficiency of calories production, given reproductive output is above 1 (upper panel) and herd growth and reproductive output (lower panel).

**Supp. Fig. 8**

Milk and MOW calories per feed consumed for simulations following the sex-asymmetric model. Main panel shows sustainable strategies (i.e. herd growth >1) only. Inlay shows all simulated datasets without constraining to herd growth >1, where line indicates equal economic efficiency of milk and meat calorie production.

**Supp. Fig. 9**

Comparison to inferred productivity and herd growth between Early Neolithic (left panel) at later Neolithic sites (right panel b; see Results for details).
